# Mechanisms of amyloid formation revealed by solution NMR

**DOI:** 10.1016/j.pnmrs.2015.05.002

**Published:** 2015-05-27

**Authors:** Theodoros K. Karamanos, Arnout P. Kalverda, Gary S. Thompson, Sheena E. Radford

**Affiliations:** Astbury Centre for Structural Molecular Biology and School of Molecular and Cellular Biology, University of Leeds, Leeds LS2 9JT, United Kingdom

**Keywords:** NMR spectroscopy, Amyloid fibrils, Invisible states, Intrisically disordered proteins, Dynamic interactions

## Abstract

Amyloid fibrils are proteinaceous elongated aggregates involved in more than fifty human diseases. Recent advances in electron microscopy and solid state NMR have allowed the characterization of fibril structures to different extents of refinement. However, structural details about the mechanism of fibril formation remain relatively poorly defined. This is mainly due to the complex, heterogeneous and transient nature of the species responsible for assembly; properties that make them difficult to detect and characterize in structural detail using biophysical techniques. The ability of solution NMR spectroscopy to investigate exchange between multiple protein states, to characterize transient and low-population species, and to study high molecular weight assemblies, render NMR an invaluable technique for studies of amyloid assembly. In this article we review state-of-the-art solution NMR methods for investigations of: (a) protein dynamics that lead to the formation of aggregation-prone species; (b) amyloidogenic intrinsically disordered proteins; and (c) protein–protein interactions on pathway to fibril formation. Together, these topics highlight the power and potential of NMR to provide atomic level information about the molecular mechanisms of one of the most fascinating problems in structural biology.

## 1. Introduction

**Fig. 1:**
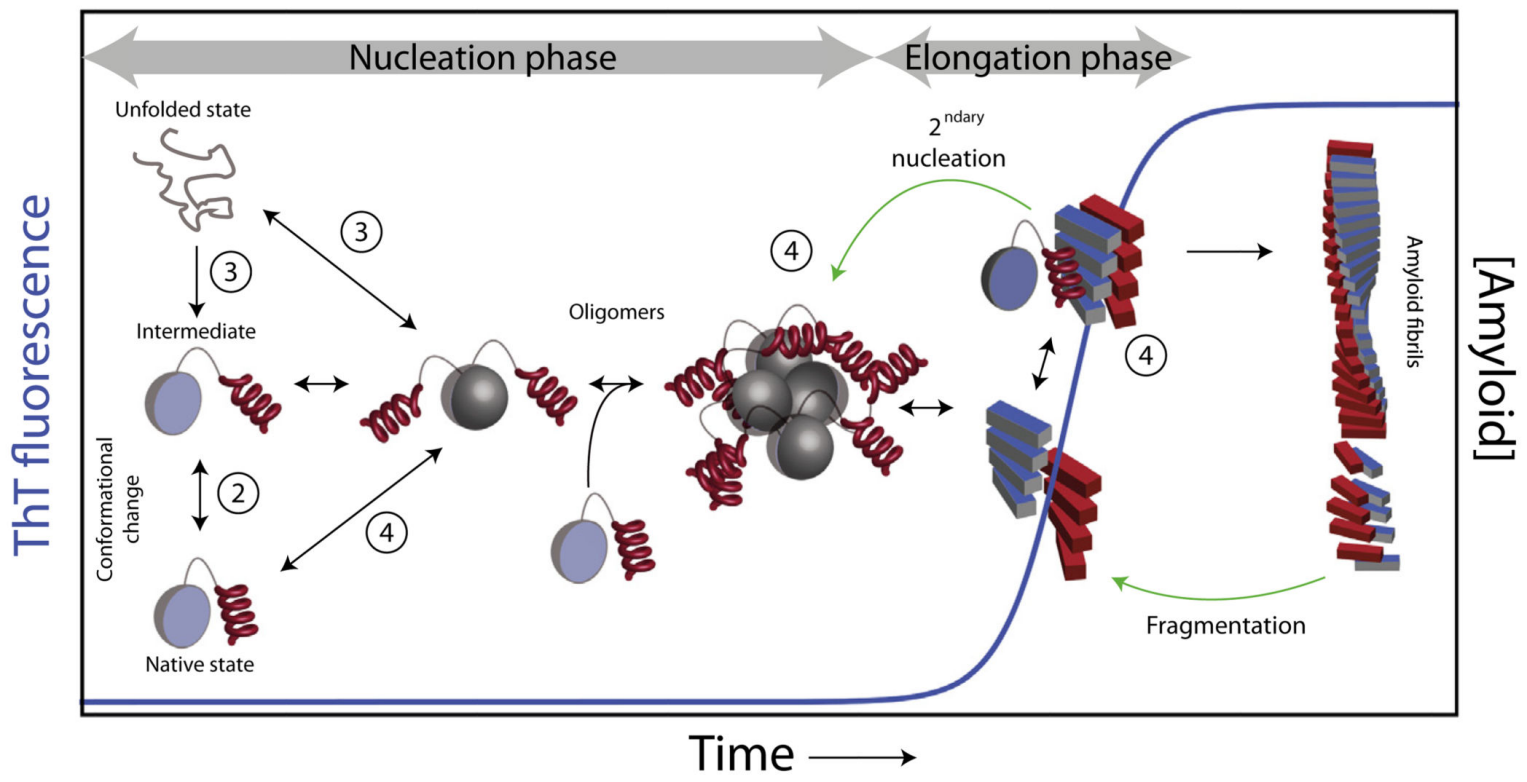
Mechanisms of amyloid assembly. The schematic represents some of the possible routes of amyloid formation through primary (black arrows) or secondary pathways (green arrows). Assembly commences from a monomeric precursor that could be unfolded, partially folded or natively folded (left-hand side). During the nucleation phase, the dynamic equilibrium between these states is responsible for generating species with increased amyloid potential, which then self-assemble. Once the critical nucleus is generated rapid formation of b-rich amyloid fibrils starts (elongation phase). Secondary mechanisms, such as secondary nucleation on the surface of preformed fibrils (or aggregates), or fibril fragmentation, are also crucial determinants of the fate of assembly [4,5,10–13]. The fluorescence of thioflavin T (ThT-blue trace), a dye that binds to cross-β aggregates, is commonly used to follow the progress of the reaction. The circled numbers denote which part of the cascade each section of this review describes.

Amyloid diseases are devastating disorders caused by the polymerization of initially innocuous proteins into amyloid fibrils[1–3]. Amyloid assembly is a multistage process in which complete or partial unfolding of the monomeric precursor is usually (but not always) the triggering event [4,5], generating aggregation-prone species. Alternatively, amyloid assembly may commence from intrinsically disordered peptides or proteins [1]. These amyloidogenic (partially) unfolded monomers are then incorporated in a cascade of protein–protein interactions until the formation of the critical nucleus (nucleation phase, Fig. 1), in a process reminiscent of the early stages of crystallization [6,7]. Later in assembly, after the formation of the critical nucleus, a second conformational change causes the assembling protein to adopt the characteristic cross-β fold of amyloid [1–3,8,9] in a rapid process (elongation phase, Fig. 1). Thus, amyloid assembly (which is commonly monitored by the fluorescence of thioflavin T (ThT) – Fig. 1) is the outcome of rare conformational rearrangements combined with heterogeneous protein–protein interactions that are abnormally interlinked.

Despite the fact that the general features of the amyloid fold (fibrillar morphology and cross-β architecture) shared by all amyloid fibrils were first discovered in the 1950s and 1960s using electron microscopy (EM) [1,14,15] and fiber diffraction studies [6–8,16–18], the precise structural and mechanistic details that govern amyloid formation remain largely elusive. Recent developments in the fields of cryo-electron microscopy, electron tomography and solid-state NMR spectroscopy as well as the use of small peptide fragments in X-ray studies, have allowed the characterization of the cross-β structure of amyloid in near atomic resolution [19–26]. However, the transient and heterogeneous nature of the monomeric and oligomeric species present during assembly render solution NMR the only technique for characterizing these in atomic detail.

In this article we review the contribution that state-of-the art solution NMR methods have made to the investigation of the mechanisms of amyloid assembly using examples obtained in the authors’ own laboratory as well as those of others. The review is organized in three parts. After briefly mentioning the challenges in studying amyloidogenic proteins by NMR, we move on to discussing the NMR methods used to characterize the conformational dynamics of the native state that lead to the population of aggregation-prone intermediates. The second part of the review focuses on intrinsically disordered proteins that aggregate into amyloid fibrils and how NMR has been used to study their structural flexibility that drives amyloid formation. To finish, NMR approaches that can be used to study the protein–protein interactions formed in early amyloid precursors and how these species subsequently form higher-ordered species, important for primary or secondary processes (such as secondary nucleation events), are discussed. Overall, the review demonstrates how NMR is contributing to developing a new understanding of every stage of the amyloid cascade at a structural molecular level.

### 1.1. Challenges in studying aggregation-prone proteins by NMR

NMR studies usually need to be performed on relatively highly concentrated protein samples (0.1–1 mM). For soluble proteins, increased concentration results in improved signal-to-noise ratio, but when studying aggregation-prone proteins, elevated protein concentrations are likely to result in enhanced oligomerisation that can complicate analysis and/or interpretation of an NMR experiment. Self-association also leads to an increase in the effective correlation time for molecular tumbling, causing the NMR signals to become broader or undetectable. Since aggregation is highly dependent on protein concentration and solution conditions, it is important to balance the protein concentration and the experimental acquisition time in order to optimize the sample for analysis by different NMR techniques. Sample quality can be tested at the beginning and the end of each experiment (for instance by recording ^1^H-^15^N HSQC fingerprint spectra). Another avenue is to fine-tune the experimental conditions to modulate the aggregation propensity. In this regard, NMR spectra of amyloidogenic proteins are often assigned under non-amyloidogenic conditions and the chemical shifts are then transferred to the more relevant (aggregation-prone) conditions via titration experiments [27–29]. The need to ensure that the starting material is purely monomeric and is not contaminated by even small amounts of aggregates or oligomers is of particular importance. For instance, when analyzing the conformational dynamics of the native state that may lead to the population of aggregation-prone species, in the absence of an appropriate kinetic model the presence of even small amounts of oligomers can make the data misleading. In such cases, complementary techniques such as analytical ultracentrifugation (AUC), dynamic light scattering (DLS), analytical size exclusion chromatography (SEC), fluorescence correlation spectroscopy (FCS), or short NMR TRACT or diffusion experiments [30] can be used to ensure the quality of the starting material.

## 2. Studying intermediate species that act as amyloidogenic precursors

The appearance of one or more partially structured states that are prone to oligomerization, or become partially structured upon oligomer formation is key to amyloid assembly, as depicted in Fig. 1. Such species can be formed from the natively folded protein, or from the unfolded (or intrinsically disordered) state. The following two sections describe NMR methods that can be used to investigate the very first steps of oligomerization and subsequent fibril assembly starting from the native state (Section 2) or from the unfolded state (Section 3).

In cases where the natively folded protein is not amyloidogenic but is in exchange with an amyloidogenic state, the population of these species is often highly skewed toward the native state. However, as long as different species are in exchange with each other on a suitable timescale, a number of NMR methods can be utilized to obtain structural, kinetic and thermodynamic information about the aggregation-prone species. These methods are highly sensitive to the properties of the ‘hidden’ (lowly populated) state, while detection takes place via the major, highly populated state.

### 2.1. Conformational dynamics investigated by relaxation-dispersion NMR

**Fig. 2:**
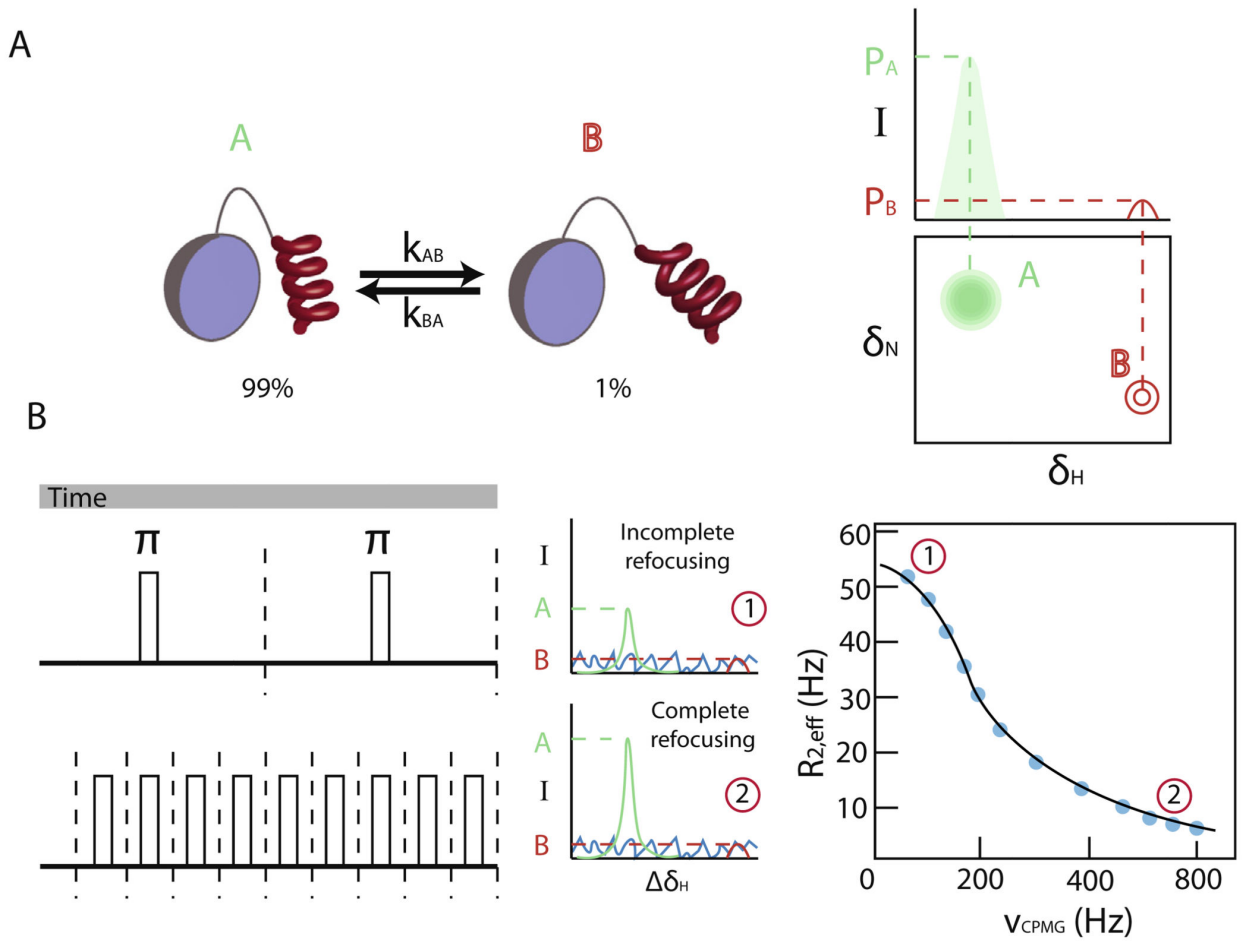
Relaxation dispersion CPMG experiments. (A) In a slowly exchanging system between a major state A and a minor state B the corresponding NMR spectrum should show two peaks, assuming that state A and state B show a chemical shift difference, and that the rate of exchange *k*_ex_ < Δδ (*k*_ex_ = *k*_AB_ + *k*_BA_). The peak intensities are then proportional to the populations of each state. However, in a system like the one shown, where the populations are highly skewed toward state A, the peak corresponding to state B is likely not to be detectable (no noise is shown in the spectra for clarity). (B) When a refocusing 180° pulse is applied the spin will be refocused after a period of time *t* (marked as 2), unless exchange is taking place during that time interval. If exchange is active, the refocusing is not complete, resulting in a loss of signal intensity due to line broadening (marked as 1). When a small number of refocusing pulses is applied (top), the chances for complete refocusing are lower since the time between the pulses is longer, allowing for more exchange events to happen. In a CPMG experiment the peak intensity is measured against the frequency of the refocusing pulses resulting in a typical profile shown in the bottom right-hand side. *P*_A_ and *P*_B_ denote the populations of states A and B respectively, which are proportional to the peak intensity.

As depicted in Fig. 1, the initiating event of protein aggregation in many cases involves conformational exchange between the non-amyloidogenic native state (A) and a highly amyloidogenic intermediate state (B) (Fig. 2A). The populations of these species are often highly skewed toward the native state and thus interconversion takes place between a highly populated, ground state A, and a lowly populated, excited state B. If the exchange is slow on the NMR chemical shift timescale (where the rate of exchange *k*_ex_ = *k*_AB_ + *k*_BA_, is slower than the chemical shift difference between the species Δδ, *k*_ex_ < Δδ) then two separate peaks should be visible in the NMR spectrum (assuming that the two spin states develop different chemical shifts) (Fig. 2A). However, if the populations of the inter-converting species are severely skewed toward one of them (the main species), then direct detection of the minor peak can be difficult or impossible [31,32]. In this instance, it is possible to detect the chemical shift of the minor state by observing changes in the intensity (or line shape) of the main peak. The basic idea behind relaxation dispersion experiments is to quantify the effective line width of peaks in the spectra as a function of the number of refocusing pulses in a Carr–Purcell–Meiboom–Gill (CPMG) pulse sequence [33,34]. Parameters that can be extracted from such an experiment include *k*_ex_ (from which the populations of the conformations in equilibrium can be calculated), but also the chemical shifts of the excited states. This then enables structural characterization of the lowly populated species which cannot be obtained in atomic resolution by any other technique [35–38].

For relaxation dispersion experiments it is crucial that the probes (spins) evolve at different frequencies (chemical shift) in states A and B (noted by ω_A_ and ω_B_, respectively). If we consider a period of time, *t*, where the spin exists only in state A and a refocusing pulse is applied in the middle of this period then the frequency evolution of the spin due to its chemical shift over this time is zero and the chemical shift is ‘refocused’. On the other hand, if during time *t* there is exchange (one or more events) between states A and B, the refocusing pulse is not able to fully refocus the chemical shift. In such a scenario, at the end of the pulse scheme the spin would have evolved at a frequency which depends on the time it spends in each state and the number of exchange events. If the frequency of the refocusing pulses increases, then there is higher probability for complete refocusing after each pulse, resulting in sharper peaks (Fig. 2B). By measuring the intensity of the peaks as a function of the frequency of the refocusing pulses a relaxation dispersion profile is obtained (Fig. 2B).

The recent development of pulse schemes able to detect relaxation dispersion data for a variety of (backbone) nuclei (^15^N, ^1^HN, ^13^CO, ^13^Cα and ^1^Hα) [37] combined with methodologies that allow the accurate determination of the three dimensional (3D) structure based solely on chemical shifts [39], allows the characterization of the ‘invisible’ species in atomic detail [31]. Following such an approach (supplemented by relaxation dispersion-derived residual dipolar couplings (RDCs) and chemical shift anisotropies – see Section 3.3), Kay and co-workers were able to solve the backbone structure of an excited state of a mutant of the Fyn SH3 domain which aggregates to form amyloid-like fibrils [40,41]. The structure of the intermediate shows a native-like arrangement of the main-chain apart from the N- and C-terminal regions. These minor differences are enough to expose hydrophobic parts of the protein that presumably act as nucleation points for amyloid aggregation [40]. CPMG studies on the human immunoglobulin κIV light-chain variable domain (LEN) helped to reveal that motions on the slow timescale (ms), related to intermolecular interactions across the interface of the dimer, are correlated with amyloid formation [42]. At near physiological pH (6.0), conditions under with the protein is not amyloidogenic, minor CPMG profiles were observed (population of the excited state ~2–4%) suggesting that the protein is stable in its dimeric form. In contrast, when the pH was decreased to 2.0 (conditions under which the protein retains a largely native fold, but becomes amyloidogenic) significant CPMG profiles were obtained for the majority of the residues in the dimer interface. The population of the excited state is increased to ~10–15% and the protein readily forms amyloid fibrils. Although the motions in the fast (ps–ns) timescale remain unchanged between the two conditions (pH 2.0–6.0), the CPMG studies showed that increased dynamics on the slow timescale (ms) are important in destabilizing the native fold, leading to dimer dissociation and amyloid assembly [42]. Relaxation dispersion was also used to reveal information about the nature of the conformational changes in the amyloidogenic monomer of transthyretin after its dissociation from the native tetramer [43].

Extension of the relaxation dispersion methodology to include side-chain atoms may lead to a full atom 3D representation of invisible amyloidogenic intermediates, and may also be used to shed light on the specific side-chain motions that are responsible for ‘native-like’ aggregation [5,44]. However, CPMG experiments have certain limitations. Exchange has to take place on an appropriate timescale (although efforts have been made to extend the range of timescales amenable to CPMG [45]) and a very high signal-to-noise ratio is required. Therefore, CPMG experiments on large macromolecular systems or proteins that aggregate too rapidly at high concentrations are difficult, but not necessarily impossible [46].

### 2.2. Characterization of amyloidogenic intermediates using real-time NMR

The folding or unfolding transitions that lead to the population of an amyloidogenic precursor can be as slow as several minutes. In these cases the kinetics of the reaction can be studied in real-time in the NMR tube [47]. To enable these studies, the unfolded protein is usually separated from the refolding buffer by an air bubble and refolding is initiated by an injection system in the NMR probe. Historically, such experiments were recorded as one dimensional data [48,49]; however recent developments in pulse sequences and data processing have allowed two dimensional (2D) and three dimensional (3D) experiments to be acquired in seconds, or even relaxation measurements in real-time [50–52].

**Fig. 3:**
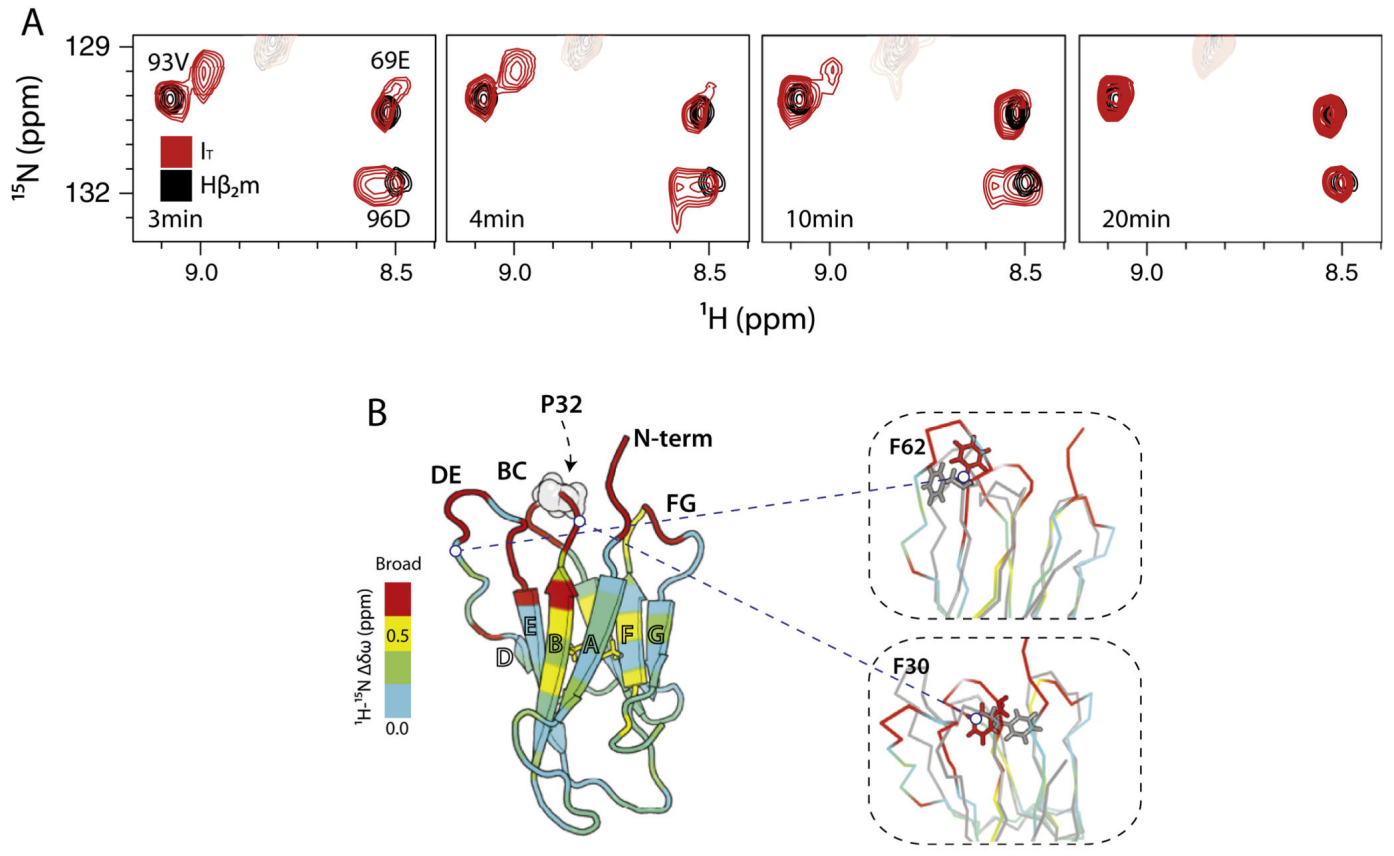
Real-time characterization of protein folding. (A) Regions of ^1^H-^15^N SOFAST-HMQC spectra recorded during the folding of human β_2_m (in 10 mM sodium phosphate buffer pH 6.2, 25 °C, 0.8 M final urea concentration). Resonances for 93V, 69E and 96E are shown in a series of spectra recorded at 3, 4, 10, 20 min after folding was initiated by 10-fold dilution of a protein solution containing 8 M urea (acquisition time 45 s). Black – native β_2_m, red – real time spectra of the *I*_T_ state that is populated en route to the native state. (B) Ribbon representation of human β_2_m colored according to the chemical shift differences between the native and *I*_T_ states. Residues that locate near P32 (that undergoes Pro isomerization) are broadened beyond detection in the spectrum of *I*_T_ (red), suggesting that they undergo significant conformational changes during folding. To illustrate the re-arrangements in the apical part of the molecule, F30 and F62 are shown as sticks on human β_2_m (red) and ΔN6 (gray) in the inset [69].

The main condition for monitoring reactions by real-time NMR experiments is that the transition must be slow, in the range of seconds to minutes. Beta-2 microglobulin (β_2_m), the light chain of the major histocompatibility complex I (MHC-I) [53,54], accumulates as amyloid fibrils in the joints of patients undergoing haemodialysis, giving rise to the disorder dialysis-related amyloidosis [55–59]. β_2_m folds slowly, which is attributed to isomerization of a single proline residue (Pro 32) [60–62]. As a consequence of the large energy barrier for proline-isomerization (70–80 kJ/mol) an intermediate containing a non-native trans isomer at position 32 accumulates (termed *I_T_*) [62,63]. Under native-conditions, the concentration of *I_T_* is directly related to the aggregation propensity of the protein (fibril elongation rate) [62] and its structural characterization was crucial for understanding the aggregation pathway of β_2_m [27,64]. Real-time NMR studies have shown that residues in the apical region of the protein, in close proximity to Pro32, undergo slow conformational dynamics, while the rest of the protein retains a native-like structure [65,66]. Comparison of the chemical shifts of the *I_T_* state, collected in real-time (Fig. 3A), with those of the truncation mutant ΔN6, in which the N-terminal 6 amino acids are deleted, confirmed the structural similarity of the two proteins [27]. Structural characterization of the aggregation-prone ΔN6 by solution NMR [27] and X-ray crystallography [64] confirmed that the protein contains a trans peptidyl X-Pro 32 bond and has an otherwise native-like structure. However, Pro isomerization causes major rearrangements of the hydrophobic core, resulting in exposure of previously buried hydrophobic residues, a phenomenon which leads to an increased aggregation potential for this β_2_m variant (Fig. 3B) [67,68].

Real-time NMR studies on α-lactalbumin, another protein able to form amyloid-like fibrils (although not connected to disease), allowed the characterization of its folding pathway. α-lactalbumin is known to populate a molten globule state [70], which is stabilized at low pH (2.5). Molten globules are characterized by the presence of native-like secondary structure, but lack persistent tertiary structure, making them vastly heterogeneous ensembles, difficult to characterize by conventional NMR techniques. Precise determination of the folding rates for the majority of residues of α-lactalbumin by 1D [49] and 2D real-time NMR showed similar time constants for all residues, revealing that the transition from the molten globule to the native state is two state [71]. Importantly, the flexibility and the precise structural characteristics of α-lactalbumin’s molten globule states, generated under different conditions (low pH or removal of the Ca^++^ ions bound to the protein) dictate the rate of fibril assembly, suggesting that the formation of a flexible/unfolded state is required for successful amyloid assembly [72].

### 2.3. H/D exchange studies on folded amyloid precursors

H/D exchange experiments provide a powerful tool for the characterization of local and/or global structural fluctuations of native proteins (native state H/D exchange) that take place on a timescale of milliseconds or slower [73–75]. These motions may expose sequences with increased amyloid potential and act as triggers of aggregation (Fig. 1). The combination of H/D exchange with the residue-specific information available using NMR spectroscopy thus offers the possibility of a detailed characterization of these aggregation-prone species.

In a H/D exchange experiment, numerous parameters affect the rate of exchange such as temperature, pH and the amino acid sequence [76]. Hydrogen atoms in native proteins are protected from exchange with deuterons by the formation of hydrogen bonds and/or by burial from solvent, implying that a global or local ‘opening’ event has to occur prior to H/D exchange. Scheme (1) summarizes the H/D exchange reaction in a native protein [74,76]:

(Scheme 1) $${\mathrm{NH}}_{\text{closed}}\overset{k_{\mathrm{op}}}{\underset{k_{\mathrm{cl}}}{↔}}{\mathrm{NH}}_{\text{open}}\overset{k_{\mathrm{ch}}}{↔}\mathrm{ND}$$

where *k*_op_ is the rate of protein opening (unfolding), *k*_cl_ is the rate of protein refolding and *k*_ch_ is the intrinsic rate of H/D exchange from the open state. As is evident from Scheme (1) the observed rate of exchange (*k*_obs_) upon exposure of a protonated protein in a deuterated buffer is given by:

(1) $$k_{\mathrm{obs}}=\frac{k_{\mathrm{op}}k_{\mathrm{ch}}}{k_{\mathrm{op}}+k_{\mathrm{cl}}+k_{\mathrm{ch}}}$$

Assuming that for a natively folded protein the rate of opening is much slower than the rate of closing, Eq. (1) can be simplified as follows:

(2) $$k_{\mathrm{obs}}=\frac{k_{\mathrm{op}}k_{\mathrm{ch}}}{k_{\mathrm{cl}}+k_{\mathrm{ch}}}$$

Based on Eq. (2) different exchange mechanisms emerge depending on the relationship between *k*_cl_ and *k*_ch_. When the rate of closing is much faster than the intrinsic rate of H/D exchange (*k*_cl_ >> *k*_ch_) Eq. (2) translates into:

(3) $$k_{\mathrm{obs}}=\frac{k_{\mathrm{op}}k_{\mathrm{ch}}}{k_{\mathrm{cl}}}=K_{\mathrm{op}}k_{\mathrm{ch}}$$

This mechanism of H/D exchange is known as EX2 exchange and usually is the outcome of small, non-cooperative fluctuations in native fold. Importantly the determination of the equilibrium position *K*_op_ allows the calculation of the free energy of unfolding. The opposite scenario where *k*_cl_ << *k*_ch_ allows the removal of *k*_cl_ from Eq. (4), resulting in EX1 exchange:

(4) $$k_{\mathrm{obs}}=k_{\mathrm{op}}$$

Thus, EX1 exchange reports on the kinetics of the unfolding process, which is usually the outcome of large scale motions, or results from the protein visiting the globally unfolded state. The protection factor (PF) is a metric of how much more slowly the exchange is taking place in the folded protein structure in comparison with the same sequence in an unfolded polypeptide chain and is defined as:

(5) $$\mathrm{PF}=k_{\mathrm{ch}}∕k_{\mathrm{obs}}$$

When analyzed by NMR, the folded protonated protein is introduced into a deuterated buffer and the peak intensities (normally of amide protons) are measured over time. Peak intensities are traditionally followed by one dimensional or 2D NMR methods [77,78]. More recently, fast-pulsing sequences [51] or non-uniformly sampled pulse schemes [52] have been used to improve temporal resolution, such that rapidly exchanging amides (minutes to hours) can also be monitored. Fitting of the intensity profiles to a single exponential function yields *k*_obs_, while *k*_ch_ can be approximated based on the primary sequence and the solution conditions using software tools such as CIntX (http://sblab.sastra.edu/cintx.html) [79]. Another approach involves the transfer of the magnetization from the protein under investigation to water molecules through H/D exchange. This methodology is particularly suited to very rapidly exchanging systems (sub-second), since pulse schemes such as CLEANEX-PM [80,81] can achieve the magnetization transfer with millisecond time resolution.

Owing to the power of the method and its relative ease of use, H/D exchange has been used to study protein stability and protein folding of many proteins in both kinetic and equilibrium experiments [73,76]. Other applications include the investigation of protein dynamics [76], the identification of binding interfaces [82], and the characterization of allosteric effects upon binding[83,84]. In terms of protein misfolding, numerous studies have been performed on amyloid precursors including Aβ40/42[85,86], α-synuclein [87], β_2_m [84,88,89], lysozyme [90,91], prion protein (PrP) [92] and transthyretin [93]. For an extended review of H/D exchange methods to study amyloid aggregation see Ref. [94]. A general correlation between a loss in H/D exchange protection and increased amyloidogenicity is suggestive of partial unfolding of the natively folded protein as the initial step that triggers aggregation. Especially in the case of β_2_m, transient intermolecular interactions are responsible for destabilizing the native fold (as monitored by H/D exchange), which catalytically converts non-aggregation-prone molecules to amyloid precursors by biomolecular interactions [84]. Importantly, the recent development of chemical exchange saturation transfer (CEST) pulse sequences allows the determination of H/D exchange rates even in lowly populated excited protein states [95]. Apart from studying (near) natively folded monomeric precursors, H/D exchange has also been used extensively to study oligomeric species and fibrils themselves. In this case the kinetic models and the experimental setup differ and will be discussed later (Section 4.3).

## 3. The role of disorder in amyloid aggregation

Proteins exhibit motions that span a wide range of timescales (from ns to mins or slower) [96,97]. These motions have been connected with various aspects of protein function, such as enzymatic catalysis [98], regulation of protein synthesis [99] and many others [100]. Proteins that possess at least one region that does not have a fixed secondary or tertiary structure are known as intrinsically disordered proteins (IDPs) and lie at the extreme of the spectrum for molecular motions, since their disordered regions can sample a very large range of different conformations[101,102]. IDPs are often found in systems where transient interactions are desirable, or systems where the interactions have to be switched on or off quickly, such as cell signaling [103,104]. IDPs also have various other advantages in comparison with their folded counterparts. Upon binding they can adopt conformations that are sterically hindered for structured proteins, such as wrapping around their protein partner in order to maximize the buried surface area [105,106]. From an evolutionary point of view, disordered regions are less likely than folded proteins to lose binding capacity or functionality upon mutation [105,107] and can act as mediators of shuffling protein domains [108]. As an outcome, IDPs can access functional mechanisms that are not available to their folded counterparts and, therefore, are advantageous for evolution. Based on all these characteristics, IDPs can promote splicing, expose or hide binding interfaces, or promote protein modularity, explaining their prevalence in the eukaryotic kingdom (more than 25% of the proteome) [109].

In the early days of studies of amyloid formation using biophysical approaches, aggregation was commonly carried out under non-native conditions, such as low pH or in the presence of co-solvents such as sodium dodecyl-sulfate (SDS) or tri-fluoroethanol (TFE) [110–112]. These studies led to the discovery that all (or most) proteins can form amyloid-like fibrils from their unfolded/disordered state under the appropriate conditions [3]. Proteins that are natively disordered are associated with numerous amyloid diseases, including Aβ (Alzheimer’s disease), α-synuclein (Parkinson’s), tau protein (dementia) and amylin (type II diabetes) [113]. Due to their heterogeneous nature and the fact that they sample such an enormous conformational space, NMR has a significant advantage for the characterization of disordered proteins, as each NMR observable represents the (per residue) ensemble average over all available conformations. On the other hand, the numerous degrees of freedom of the system require the measurement of a large number of uncorrelated complementary restraints in order to reach convergence [114]. Fortunately, NMR chemical shifts, paramagnetic relaxation enhancement (PRE) and residual dipolar couplings (RDCs) are all independent measurements that can be used to describe the complex structural behavior of IDPs. The next sections give a brief overview of how these NMR methods can be used to investigate these structurally dynamic proteins.

### 3.1. Chemical shifts probe transient secondary structure propensity

The chemical shift reports accurately on the nature of the electronic micro-environment of each nuclear spin, and thus chemical shifts are inherently sensitive to changes in the primary sequence, secondary/tertiary structure, ligand binding, protein–protein interactions and many other biologically relevant phenomena. Early on, it was realized that the chemical shift can be used to infer structural information about proteins [115] by comparing the deviation of experimentally measured ^1^H and ^13^C chemical shifts with respect to their random coil values (secondary chemical shift – δ_SCS_). The δ_SCS_ is a metric often used to identify the secondary structure content of a protein as it is easy to determine and reliable. However, δ_SCS_ depends on the definition of the random coil chemical shift values, a quantity that remains to be precisely defined and depends on the model used for a random structure, the amino acid sequence, the temperature and the pH [116–118]. This sensitivity to the precise reference conditions is particularly important when interpreting chemical shifts of disordered proteins.

Since the chemical shift is so sensitive to a variety of factors it can be difficult to deconvolute the true contribution of structure on the observed shift, especially for proteins that do not have a persistent fold such as IDPs. However, with the increasing number of available native protein structures whose chemical shifts are known experimentally, it has become possible to develop machine-learning approaches to predict the chemical shift directly from the three-dimensional structure of a protein. These approaches include SHIFTX^2^ [119], SPARTA+ [120] and CamShift[121], all of which rely on a static representation of the protein. Recently, a method that takes protein dynamics into account by predicting (the potentially already ensemble-averaged) chemical shifts during long molecular dynamics (MD) trajectories has been developed [122–124]. However, it has to be noted that chemical shifts depend highly on the experimental conditions, making their prediction and detailed interpretation difficult in the absence of supplementary evidence, especially for IDPs and dynamic protein ensembles. Chemical shifts have been used as powerful complementary restraints alongside other NMR observables that, combined together, can provide an accurate description of the conformational distribution of amyloidogenic IDPs (see Section 3.4).

### 3.2. Paramagnetic relaxation enhancement (PRE)

**Fig. 4:**
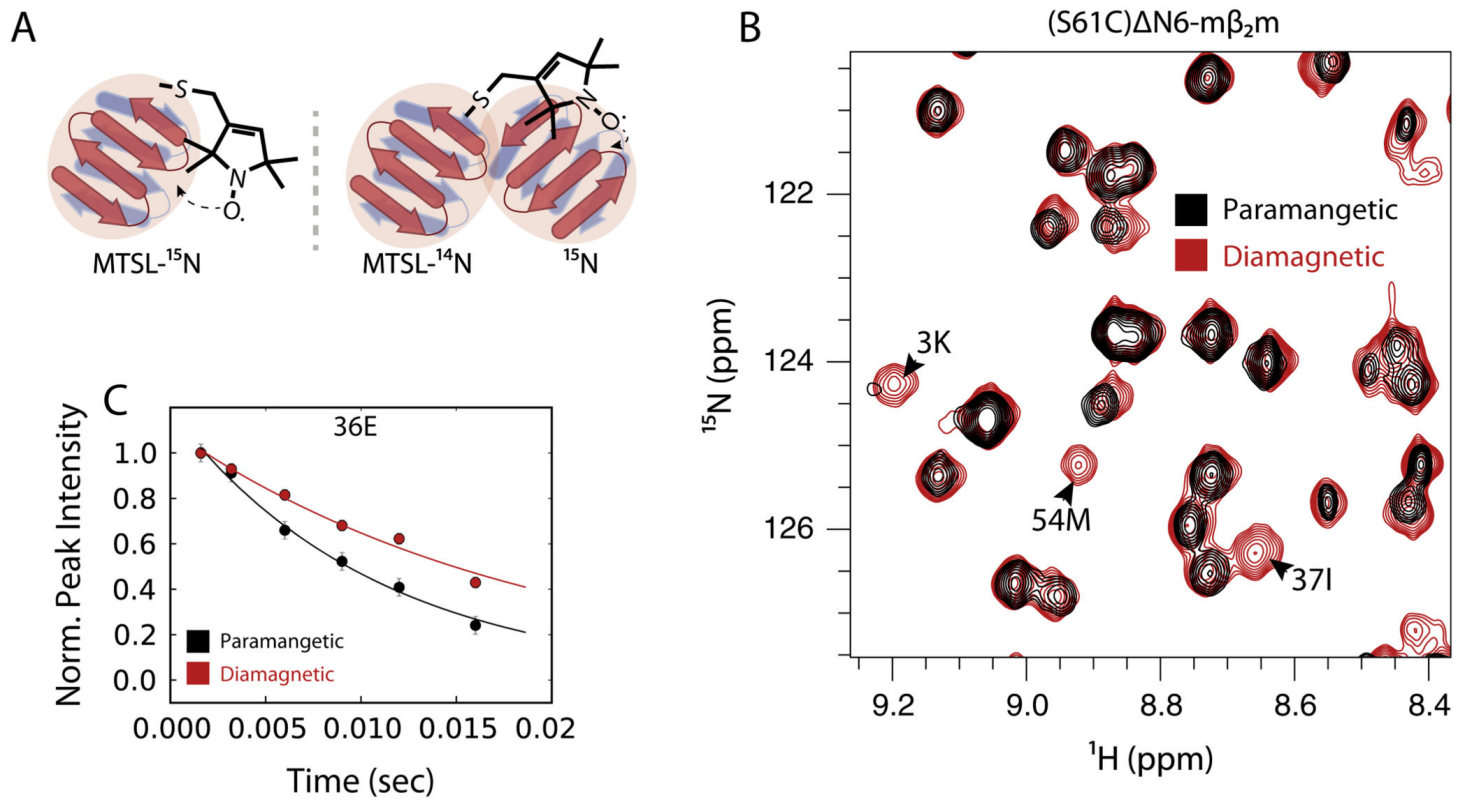
Principles of paramagnetic relaxation enhancement. (A) Types of PRE experiments (intramolecular PRE-left, intermolecular PRE-right). (B) Overlay of a region of a ^1^H-^15^N HSQC spectrum of ^15^N-labeled murine β_2_m (mβ_2_m) in presence of ^14^N-labeled ΔN6 containing oxidized (paramagnetic sample – black) and reduced MTSL (diamagnetic sample – red). Missing peaks from the paramagnetic spectrum belong to amides that locate closely to the position of the spin label. (C) ^1^H-*R*_2_ relaxation rates (paramagnetic-black, diamagnetic-red) for the E36 amide which locates close to the interface of the two proteins. Solid lines represent fits to single exponentials [69,84].

In the presence of a paramagnet such as 1-oxyl-2,2,5,5-tetram ethyl-Δ3-pyrroline-3-methyl) methanethiosulfonate (MTSL – Fig. 4A), nuclei show a different magnetic response owing to various molecular phenomena such as dipole-dipole interactions, Curie-spin relaxation and pseudocontact shifts (reviewed in [125]). The paramagnetic effect can be detected by an increased relaxation rate of nearby nuclei (PRE), pseudocontact shifts (PCS) and residual dipolar couplings (RDC) (Fig. 4). The PRE can be measured in any paramagnetic system, while PCS and RDC measurements require an anisotropic g-tensor, meaning that it is necessary to assign the paramagnetic and diamagnetic states separately, a task that can be challenging [126,127]. PRE arises from the magnetic dipolar interactions (in most cases) between a nucleus and an unpaired electron of the paramagnetic probe. While both the nOe and PRE show the same *r*^−6^ distance dependence (between the neighboring nuclei (nOe), or the nucleus with the paramagnetic probe (PRE)), owing to the large magnetic moment of the electron the PRE phenomenon extends to much larger distances (up to 35 Å), depending on the paramagnetic group used. The effect of the dipole–dipole interaction is an increase in the relaxation rates of the nuclear magnetization, which can be measured using standard relaxation pulse schemes (Fig. 4B and C). Typically, relaxation rates (normally ^1^H) are measured in the presence of a spin label and subtracted from their corresponding values when the spin label is inactive (reduced) resulting in the measurement of the PRE *Γ*_2_ rate [123]:

(6) $$\Gamma_{2}\;=\;R_{2,\text{para}}-R_{2,\text{dia}}$$

(7) $$r\;=\;{\left\{\frac{K}{\Gamma_{2}}\left(4\tau_{c}+\frac{3\tau_{c}}{1+\omega^{2}\tau_{c}^{2}}\right)\right\}}^{1∕6}$$

where *R*_2,para_, *R*_2,dia_ are the relaxation rates in the paramagnetic and diamagnetic sample respectively, *r* is the distance between the spin label and the nucleus, τ*_c_* is the correlation time of the electron–nucleus interaction (assumed to be the global correlation time of the protein for nitroxide spin labels), ω is the Larmor frequency of the spin (proton) and *K* is 1.23 * 10^−32^ cm^6^ s^−2^.

The subtraction in Eq. (6) cancels out all relaxation processes that are common in the reduced and oxidized states, and leaves the paramagnetically-induced relaxation as the only difference[128]. Alternatively, the same measurement can be performed by comparing the peak intensities in a simple HSQC-type of experiment in a sample containing the oxidized (*I*_ox_) versus the reduced (*I*_red_) spin label (Fig. 4B). The *I*_ox_/*I*_red_ ratio can then be converted to PRE rates or directly into distances. Since the direct measurement of the PRE rates requires very high signal-to-noise ratios to allow robust fitting and is more time-consuming, determination of the *I*_ox_/*I*_red_ ratio is often the method of choice, especially for proteins of higher MW (>40 kDa) [128–133]. However, this can result in biased PRE rates because of other NMR phenomena contributing to the observed peak intensity in the presence of the paramagnet (e.g. differential increase in the *T*_1_ relaxation between the oxidized and reduced samples), which are not taken into account. Spin labels are usually introduced via a single solvent-accessible cysteine residue (or a pair of cysteines) and are typically functionalized by nitroxide moieties and/or chelated metal ions (reviewed in [134]). The choice of the appropriate spin label is based on the distance range of the expected interaction and the precision required. Most of the spin labels are at least partially hydrophobic and therefore can cause artifacts especially when dealing with exposed hydrophobic surfaces of amyloidogenic proteins. Thus, when applied to aggregating proteins, PRE experiments need to be carefully designed to ensure that the paramagnet introduced does not affect protein structure, dynamics or aggregation.

When interpreting the PRE effect as a distance, special care has to be taken to account for the increased flexibility of the MTSL side chain. The problem can be taken care of by including an order parameter for the internal motions of the side chain [135], and/or averaging between multiple conformers of MTSL in ensemble molecular dynamics simulations [125,136]. Alternatively, Clore and co-workers presented a sophisticated theoretical framework to allow direct back-calculation of *Γ*_2_ rates from 3D structure, which is advantageous since it provides a tool for the full description of the PRE phenomenon in 3D space [125,135].

**Fig. 5:**
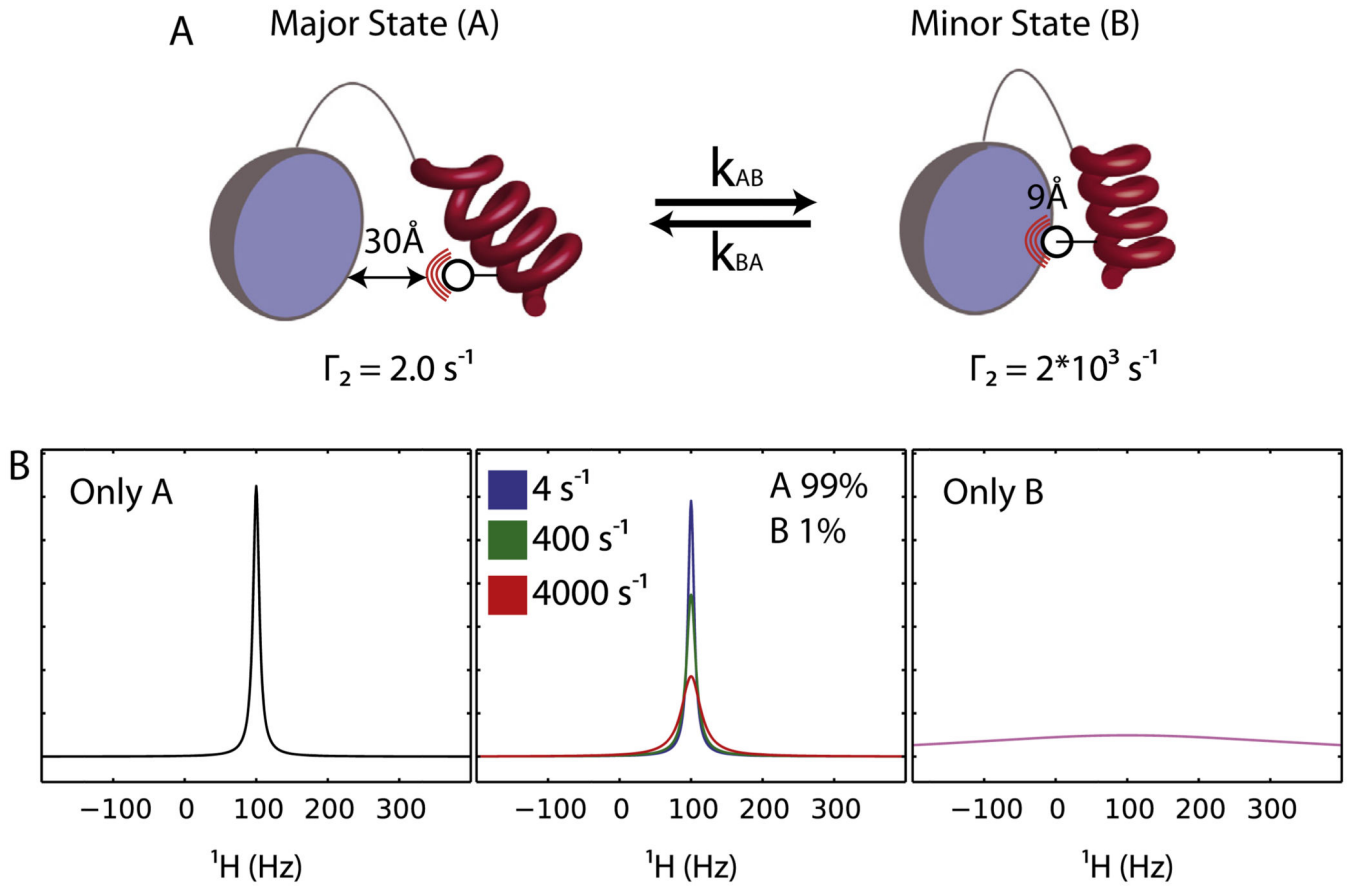
The effect of exchange rates in PRE measurements. (A) One spin-labeled (black open circle) protein that is in exchange between a major (A) and minor (B) state is shown. The extended conformation of the major state gives rise to a small *Γ*_2_ rate (2 s^−1^) for the spin label site shown, while the more compact structure of state B shows a *Γ*_2_ value of 2 * 10^3^ s^−1^. (B) Line shape simulations of the resonance of state A as the exchange rate *k*_ex_ (*k*_ex_ = *k*_AB_ + *k*_BA_) is increased from 4 to 4000 s^−1^. The simulations were performed by solving the McConnell equations for a two-state system [144], using a chemical shift of 100 Hz for both states (assuming no pseudo-contact shifts), a diamagnetic *T*_2_ relaxation rate of 30 s^−1^ and a population of 99% and 1% for states A and B, respectively. The curve of the right-hand panel is magnified by a factor of two for clarity. When the exchange rate is in the fast exchange regime the line-shape is dominated by the *Γ*_2_ rate of the minor state although its population is as low as 1%.

PRE experiments have been used to assist the structure calculation of large proteins [130], to characterize the domain organization of multi-domain systems [129,134,137,138], to study protein–DNA interactions [139–141], and to interrogate intrinsically disordered proteins [142]. However, one of the most powerful features of this technique is its ability to provide unique information about exchanging systems. In this case, the use of PRE-derived information depends highly on the timescale of exchange [143]. If the motions lie in the slow exchange regime, the PRE data can offer a significant improvement in the quality of NMR structures in comparison with the classical nOe type of calculation. On the other hand, if the timescale of exchange is fast, application of the PRE methodology allows the characterization of dynamic processes and the identification and structural elucidation of lowly populated intermediate species. More specifically, based on McConnell’s equations for a two state system, the apparent PRE rate (*Γ*_2_) is highly dependent on the rate of the exchange event (*k*_ex_ = *k*_AB_ + *k*_BA_) between states A and B (Fig. 5A). If the exchange takes place in the slow PRE regime (*k*_ex_ << *Γ*_2,B_ – *Γ*_2,A_), then the presence of the minor species B has no effect on the measured PRE $\Gamma_{2}^{\mathrm{app}}$ rate, which in this case represents only the major species A. However, for larger *k*_ex_, the $\Gamma_{2}^{\mathrm{app}}$ rate is highly influenced by the minor state B (Fig. 5A and B). Thus, when *k*_ex_ >> *Γ*_2,B_ – *Γ*_2,A_ (fast exchange regime), the apparent *Γ*_2_ rate is the weighted populated average of the *Γ*_2_ rates of the two species [125]. As IDPs are expected to interconvert rapidly between numerous conformations in a large ensemble of species, visualization of the individual ensemble members (which can be less than 1% populated) is greatly facilitated by the PRE methodology [114].

IDPs, such as α-synuclein or Aβ40/42, are expected to show some long-range interactions, much like the well-known long-range interactions of unfolded globular proteins (e.g. under denaturing conditions [145]). The presence or absence of these kinds of interactions was hypothesized to be important in the aggregation properties of these amyloid-disease related proteins. PRE studies on spin-labeled α-synuclein showed interactions between the charged C-terminal residues 120–140 and residues 30–100 in the so-called NAC region, creating a compact form of the protein [146]. This occluded conformation, as observed by molecular dynamics simulations incorporating PRE-derived restraints, was proposed to inhibit the aggregation of α-synuclein by shielding the aggregation-prone NAC region of the protein. Importantly these long-range interactions are preserved in the physiologically relevant, N-terminally acetylated version of α-synuclein [147]. Inter-chain interactions between different α-synuclein monomers have also been monitored by paramagnetic NMR. PREs were observed in the C-terminal region of the protein when the spin label was attached in the N-terminal region and vice versa, showing that a head-to-tail interaction is taking place between α-synuclein monomers [148]. However, the relatively weak PRE effect observed at neutral pH suggests that α-synuclein prefers to interact with solvent molecules rather than itself.

### 3.3. Residual dipolar couplings reveal residual structure in unfolded proteins

**Fig. 6:**
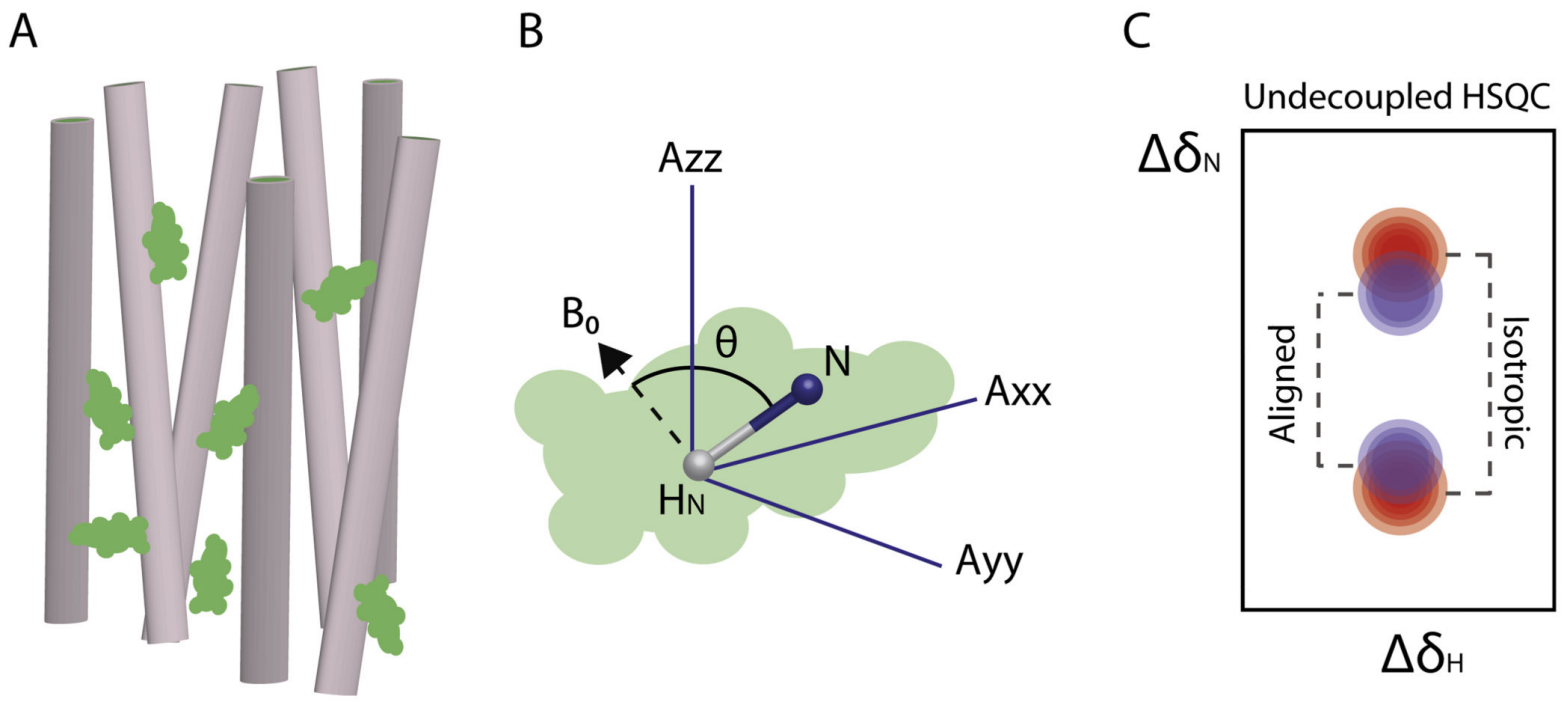
RDCs in biomolecular NMR. (A) Introduction of a protein (green) into an alignment medium (represented by the gray cylinders) partially restricts the protein’s rotational motion. (B) RDCs depend on the time-averaged angle (*θ*) between the internuclear vector (here shown as the amide bond) and the external magnetic field *B*_0_. Transformation from a vector orientation relative to the field to an orientation relative to the molecular frame gives the preferential orientational averaging of the molecule expressed as a traceless second rank alignment tensor (Axx, Ayy, Azz). The alignment tensor describes the magnitude and direction of alignment for three orthogonal orientations. (C) An undecoupled HSQC spectrum in isotropic solutions results in the measurement of the *J* coupling (red peaks). If the molecule is aligned the same experiment measures the sum of the *J* and dipolar coupling (purple peaks). Subtraction of the two gives the value of the RDC.

The through-space dipolar interaction (*D_ij_*) between two magnetically active nuclei (*i, j*) is given by:

(8) $$D_{ij}\;=-\frac{\gamma_{i}\gamma_{j}h\mu_{0}}{4\pi^{2}r^{3}}\langle \frac{3\;\cos^{2}\theta -1}{2}\rangle ,$$

where *γ_i_*, *γ_j_* are the gyromagnetic ratios of each nucleus, *h*, Plank’s constant, μ_0_ the permeability of a vacuum, *r* the distance between nuclei *i* and *j*, and θ the orientation of the nuclear vector with respect to the external magnetic field. Proteins in isotropic solutions tumble freely and thus θ can take all possible values, causing *D_ij_* in Eq. (8) to average to zero. Introduction of a protein into a medium which can be aligned with respect to the magnetic field, such as a liquid crystalline phase, causes limited restriction of the molecule’s rotation such that the dipolar coupling becomes non-zero (Fig. 6A). Measurement of the residual dipolar coupling (RDC) is extremely powerful as it contains valuable long range angular information for the internuclear vectors and can be used to complement short or long range distances measured by nOe or PRE experiments, respectively. Therefore, a variety of alignment media such as filamentous phages, stretched gels, bicelles or glycol mixtures have been developed in order to align folded proteins [149,150]. Structurally, RDCs can be analyzed as internuclear vectors oriented in a common alignment tensor that is fixed in terms of the molecular frame (Fig. 6B). For a fixed length bond between two nuclei in a rigid fully anisotropically aligned object in solution, the RDC can be calculated from the probability of alignment along three orthogonal directions in the molecules frame of reference. This information can be written as an alignment tensor. Five parameters are required to describe an RDC from a known molecular structure: three angles to align the molecule, plus the magnitude and rhombicity of alignment. For a system undergoing conformational exchange the overall time averaged RDC value can be computed from a simple weighted average of the RDC for each conformer. The important insight that allowed this formalism to be used for the ensemble modeling of IDPS is that for steric alignment it has proved sufficient to predict the alignment tensor for each of the conformations present in an ensemble of limited size as a static snapshot without reference to time variations in order to generate an empirically convincing model. Alternatively, new computational methods that do not require the calculation of an alignment tensor combined with molecular dynamics simulations may be useful in capturing the time-averaged nature of RDCs, especially for conformationally heterogeneous proteins such as IDPs [151,152]. RDCs are typically measured using inphase–antiphase sequences (IPAP) which can separate the upfield and downfield components of the *J*-coupled doublet into separate spectra (Fig. 6C) [153].

Given the extreme flexibility of IDPs, RDCs for such proteins might be expected to average close to zero even under conditions of weak alignment due to conformational averaging. However, non-zero RDCs have been measured for denatured proteins [154] showing that RDCs can be used to probe structural propensities in the conformational ensemble. H^N^-N-RDCs for IDPs show a negative sign and a bell-shape dependence on the polypeptide sequence, reflecting the propensity of the polypeptide chain to align parallel with the magnetic field [145,154,155]. However, it has been observed that RDCs in disordered proteins are very sensitive to local structure (for instance, formation of transient helices changes the orientation of the amide vector in relation to *B*_0_). Thus, the sign of the coupling and the magnitude of the observed RDC are highly correlated with the size of the side chain [156–158]. Therefore, RDCs contain valuable information about the conformational properties of a disordered polypeptide chain that reports both on the local and long-range structure.

As denoted by the angle brackets in Eq. (8), the measured RDCs report on the ensemble average over all possible conformations in solution. As an outcome, averaging of the alignment tensors of each member of an ensemble should reproduce the experimentally measured RDCs, providing that the ensemble is of adequate size and is correctly parametrized (see Section 3.4). RDC studies on α-synuclein showed that the experimentally measured RDCs could not be explained unless contacts between the N- and C-terminal regions are included in the ensemble [159]. This finding confirmed the occluded conformation of the protein as observed by PRE studies [146] and further highlights the importance of the charged residues in stabilizing these regions. Importantly, increasing the ionic strength, deletion of the C-terminal region, and/or mutation in the N-terminal region all cause increased fibrillation rates, consistent with the importance of intra-molecular interactions between the C-terminal and NAC regions, and inter-molecular interactions between the N- and C-terminal regions, during the early stages of aggregation.

Investigation of the conformational properties of the protein tau also showed large discrepancies between the measured RDCs and those predicted from the statistical coil model, in areas implicated in aggregation [160]. Accelerated molecular dynamics simulations (AMD) [161], in which the energy landscape is biased in order to favor transitions between low energy states (thus providing access to longer timescales and better conformational sampling), were used in the same study to investigate the structural plasticity of tau. This analysis revealed a high propensity for a β-turn conformation in regions of the polypeptide chain that could not be captured by the statistical coil model. Importantly, incorporation of the dihedral angle sampling derived from AMD to the coil model resulted in much better prediction of the experimentally measured RDCs [162]. These highly localized and sequence-dependent structural propensities were hypothesized to act as an inhibitor of β-rich amyloid aggregate formation [162].

### 3.4. Ensemble calculations – the importance of incorporating a plethora of data

**Fig. 7:**
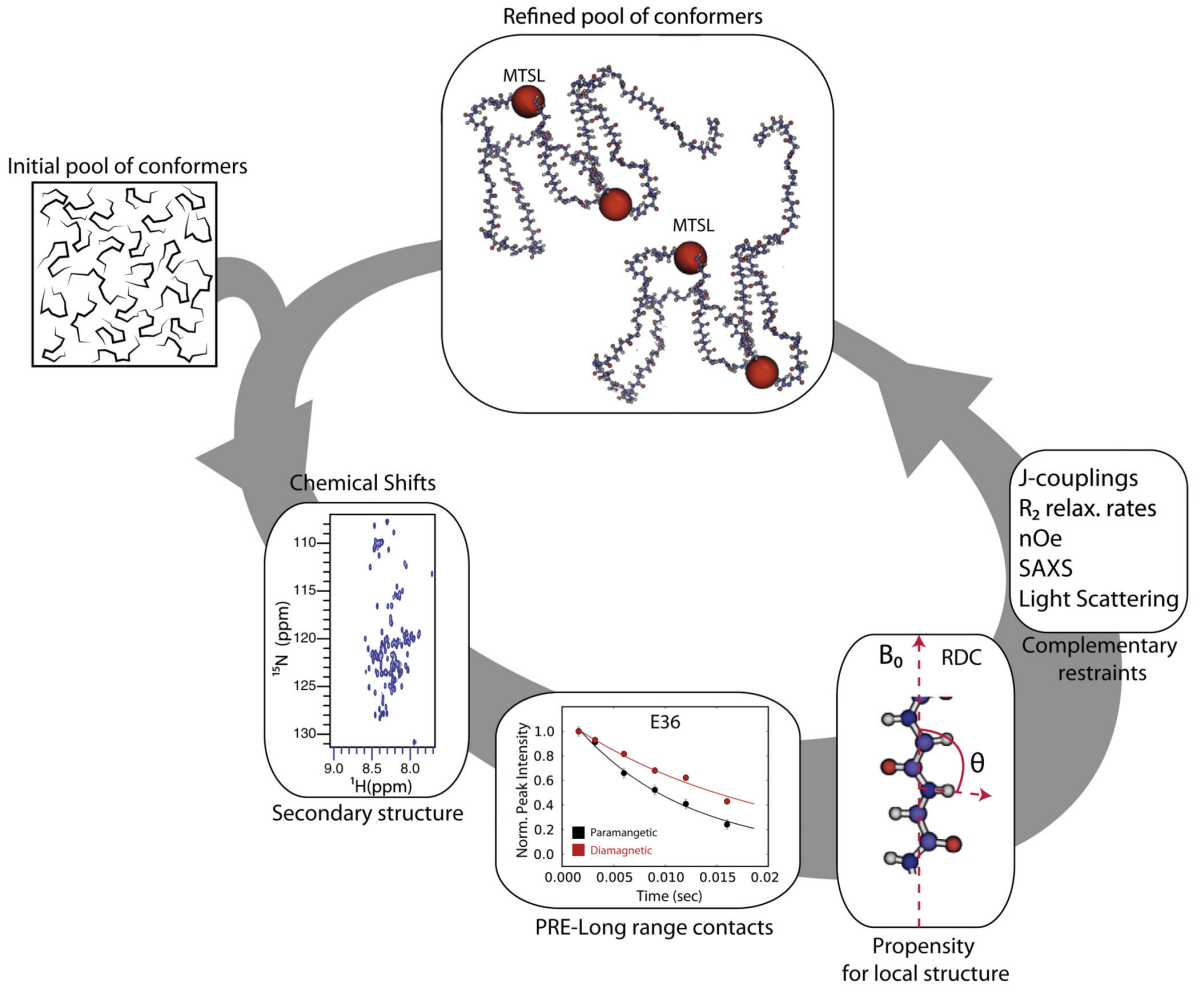
NMR strategy to study IDPs. An initial pool of conformations is generated by statistical coil approaches. This ensemble is then refined by rounds of selections against experimental restraints such as chemical shifts, PREs and RDCs. The backbone atoms of an IDP are shown in a ball and stick representation. The red spheres denote the positions where MTSL is attached, used to derive intramolecular PRE restraints (top panel). Some of the experimental restraints are usually not included in the selection, but are instead used for cross-validation of the resulting ensembles after each round of selection.

Due to the large number of degrees of freedom of IDPs, a large amount of experimental data is required to describe their conformational ensemble adequately. To resolve this issue, the available NMR restraints (chemical shifts, PREs, RDCs) can be analyzed together to generate a consistent ensemble (Fig. 7). Chemical shifts and RDCs mainly depend on local structural order, while PREs report on the long range interactions of the polypeptide chain. Combined together, these data provide a more complete representation of the conformational ensemble. In theory, chemical shift anisotropies (CSA) could also be used to aid this process; however they are difficult to measure. Originally, statistical coil models were used to generate appropriate ensembles for IDPs [114]; however these fail to reproduce large experimental datasets, since denatured proteins tend to sample extended conformations significantly [162,163]. Normally, tens of thousands of conformations need to be averaged in order to reach convergence [164], making the analysis computationally intense and time-consuming. Recent developments from Blackledge [136,164] and Forman-Kay[165] and their colleagues have allowed the generation of ensembles representative of the conformational sampling of IDPs in solution at the amino-acid level, by using statistical coil models in combination with sophisticated selection methods that allow the derivation of appropriate sub-ensembles from broader distributions [164,166] (Fig. 7). Alternatively, experimental restraints can be incorporated in restrained or replica exchange simulations[163].

Such an approach has been used in the case of α-synuclein by combining PRE and RDC data to describe the long range intramolecular interactions between the C-terminus and the NAC region in atomic details (previously observed by PRE alone) [167]. Release of these autoinhibitory interactions was hypothesized to prime the protein for aggregation [167]. MD simulations had suggested that α-synuclein adopts an ensemble containing a number of oligomeric species, some of which may be α-helical or β-sheet rich [168]. Analysis of a large experimental dataset by Blackledge and co-workers [142], including chemical shifts for five different nuclei, one set of RDCs, four sets of PRE restraints, supplemented by small angle X-ray scattering (SAXS) data, provided a better representation of the conformational behavior of the protein in solution. After careful cross-validation of the data, the unfolded nature of the protein was confirmed. In the same study, the conformational ensemble of the 441-residue full-length human protein tau was also investigated utilizing 11 sets of PREs, 5 sets of chemical shifts, 1 set of RDCs and SAXS data [142]. In both cases an elevated population for polyproline-II (βP) conformations was directly observed, especially in regions of increased amyloidogenicity, suggesting that this conformational behavior is a precursor of amyloid fibril formation. Furthermore, by combining a variety of experimental restraints, a better agreement between the experimental data and the results of MD simulation was achieved, in comparison to the statistical coil ensemble alone[142], showing the validity and the predictive power of these approaches.

## 4. Investigating interactions in higher-order species on pathway to amyloid fibrils using NMR

In the pursuit of linking the different properties of initially folded proteins to amyloidogenicity, many studies have focused on analyzing the structural properties of monomeric amyloid precursors. Unfolding energies, hydrophobicity, propensity to form inter/intra-molecular contacts and protein dynamics have all been linked to increased amyloid propensity [27,43,169]. The wealth of data available has allowed the generation of algorithms capable of predicting amyloidogenicity, based either on the physicochemical properties of the amino acid sequence involved, or the structure of the protein, and how these are modulated by the dynamics of the native state (reviewed in [170]). On the other hand, other research efforts have focused on elucidating the structure of amyloid fibrils in order to establish their role in cytotoxicity and/or protein function. The first atomic details of the structures of small amyloid-forming peptide segments were solved by X-ray fiber diffraction [171,172] and X-ray crystallography [19,24]. Structural models based on solid state NMR have also been proposed for amyloid protofilaments or fibrils, including Aβ_40_ [23,173,174], and the C-terminal domain of HET-s [21]. Very recently, the structure of an amyloid fibril involving 2, 4, or 6 protofilaments of a peptide of transthyretin was determined using a combination of solid-state NMR and EM [26].

By contrast with the highly-ordered stable amyloid fold, structural characterization of the oligomeric species formed during fibril formation has proved difficult because of the transient nature and heterogeneity of amyloid intermediates. These species are often termed ‘amyloid oligomers’ and their short lifetimes, polymorphic nature, dynamic properties and variety of mechanisms of formation make them inaccessible to most current structural tools. In addition, the distinction between off- and on-pathway oligomers, i.e. oligomers that directly precede amyloid fibrils, or represent meta-stable species on an alternative assembly pathway, remains one of the most challenging aspects of their characterization. Amyloid oligomers show increased ability to penetrate lipid bilayers, to interact with external cellular components, and to cause cytotoxicity, in comparison with monomeric or fibrillar forms of the same protein precursors, highlighting their importance in understanding the aggregation pathway and the origins of disease[175–177].

Recently, a distinction between on- and off-pathway assemblies in the early stages of amyloid formation was made possible using fluorescence techniques. Cremades et al. [178] and Campioni et al. [179] in two independent studies showed that human α-synuclein and bacterial HypF aggregate through the formation of oligomeric species of different size and structure and, as a result, have different cytotoxicity. In the former case, single molecule fluorescence techniques and the analysis of individual species in solution allowed direct observation of the inter-conversion between the different oligomeric forms. These findings highlighted the dynamic nature of protein–protein interactions in the early stages of amyloid formation, giving rise to species of the same overall shape, but different in structural details, sufficient to make them toxic (or not toxic) to the cell. Even though powerful and illuminating in that they provide insights into the overall architecture, size and shape of the oligomeric assemblies, these studies fail to reveal the structure of these key amyloid intermediates in atomistic detail. Eisenberg and co-workers reported the first high resolution crystal structure of a trapped, supposedly toxic, oligomer that appears to be on-pathway to fibrils derived from a peptide segment of αβ-crystallin [180]. Its structure revealed a novel barrel-like cylindrical fold, termed cylindrin, that is only marginally destabilised in comparison to the classic steric zipper form that amyloid peptides often adopt in crystals [24]. Interestingly, the cylindrin architecture appears to be more generic and not only related to αβ-crystallin [181].

Structurally describing the aggregates of different sizes, ranging from dimers to high molecular weight oligomers, represents a challenging task for solution NMR spectroscopy due to their size (often >1 MDa) and their heterogeneous and dynamic nature. However, significant progress has been made toward the characterization of these species, either by studying the protein–protein interactions within oligomeric species directly, or by inferring information about the interacting species by exploiting the exchange of monomeric species with the oligomers/fibrils. These new insights and the NMR approaches used are highlighted in the sections below.

### 4.1. Direct detection of oligomeric intermediates

Owing to its high sensitivity and increased chemical shift range, ^19^F NMR can be used to probe oligomer/fibril assembly directly. The position and intensity of the ^19^F signal of fluorinated proteins can provide information about the formation of oligomers in real-time during fibril assembly. Following the ^19^F signal during the aggregation of human islet associated polypeptide (IAPP) suggested that fibril assembly is a two-state process (monomer to fibrils), without the accumulation of detectable intermediate species[182]. On the other hand, at least six oligomeric species, each with a distinct NMR signature, were observed for Aβ40 [183], and the formation of an octamer was associated with enhanced amyloidogenicity of prion protein [184]. Other studies have inferred information about the kinetics of protein oligomerization from the loss of the NMR signal of the monomeric precursor during aggregation [185] while, more recently, fibril dynamics were studied directly by investigating the release of soluble oligomers and monomers using real-time NMR [186].

A new NMR technique that lies on the edge of solution and solid-state NMR approaches is the so-called SedNMR [187]. Highly concentrated solutions of high molecular weight proteins or assemblies are centrifuged either *in situ* (using the centrifugal force of the magic-angle (MAS) rotor) or *ex situ* by placing the MAS rotor in an ultracentrifuge prior to NMR analysis [187,188]. The precipitate formed on the walls of the rotor gives excellent NMR spectra in comparison with lyophilized samples or microcrystalline phases, and remains highly hydrated compared with other SS-NMR sample preparation protocols [188,189]. Therefore, SedNMR is promising for the structural characterization of soluble amyloid oligomers that are of sufficient mass to be spun out of solution, or even partially insoluble species on-pathway to fibrils. As sedimented samples contain both a solution and a ‘solid’ phase, different spectroscopic techniques can be applied to detect signals arising from each phase of the sample. Bertini et al. have applied insensitive nuclei polarization transfer (INEPT), and cross-polarization techniques, to study the solution (containing monomer and or small Aβ40 oligomers) and ‘solid’ (containing larger aggregates) phase of Aβ40 aggregates in real-time [190]. This method allowed a quantitative kinetic analysis of fibril assembly to be made and, due to the high sensitivity of the technique, assignment of the resonances of the aggregates was also possible[190].

Although SedNMR represents a promising approach for studying amyloid oligomers, the heterogeneity of these species may reduce the SS-NMR spectral quality, rendering them intractable to NMR analysis. In these cases, indirect detection of large assemblies is possible by investigating their influence on their monomeric counterparts, as described below.

### 4.2. Dark exchange saturation transfer (DEST)

**Fig. 8:**
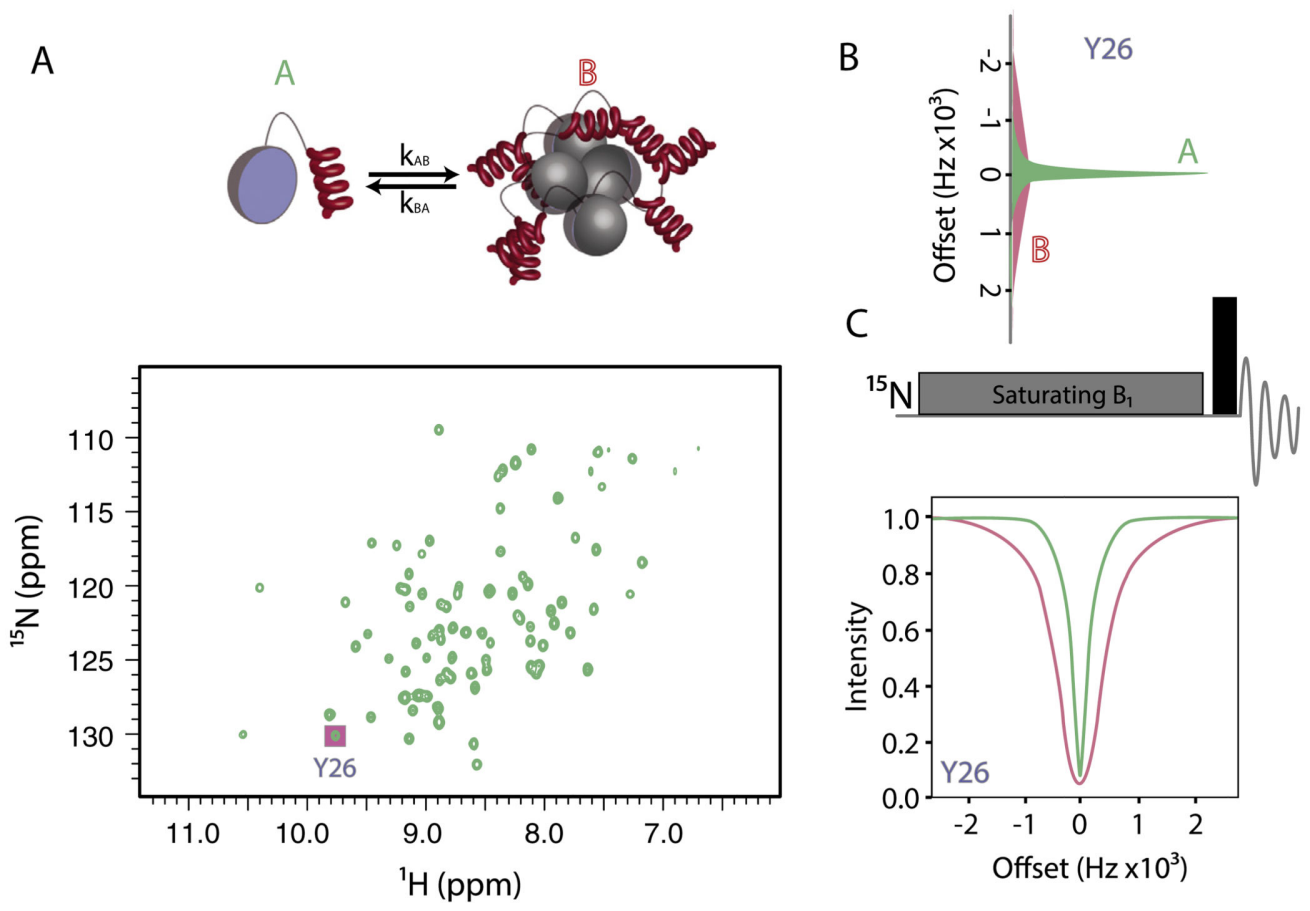
Principles of DEST experiments. (A) In a system where there is exchange between a monomeric (state A) and an oligomeric state (state B), the resulting NMR spectrum (^1^H-^15^N HSQC) shows sharp lines corresponding to the monomeric protein, while the high molecular weight species are NMR-invisible. (B) For instance, the Y26 resonance in the spectrum of human β_2_m shows a broad line arising from the oligomeric state B. (C) In a DEST experiment a weak saturating field *B*_1_ is applied at various offsets in the indirect dimension and the peak intensity on resonance is plotted against the frequency of *B*_1_. If the Y26 resonance was not in exchange *B*_1_ would have an effect only when applied on resonance (green line). However, when exchange is active, saturation is transferred from state B to A, resulting a broader saturation profile (red line).

The powerful CPMG experiments presented in Section 2.1 rely on the fact that two protein states in exchange have different chemical shifts. Based on that observation, crucial information about the minor state can be obtained by studying the modulation its presence causes on the major, visible state (as long the exchange is taking place at ~10 ms-1 s). The known limitations of solution NMR spectroscopy in resolving complexes of large macromolecular size (<1 MDa, unless the assembly contains dynamic regions) renders direct observation of a wealth of macromolecular assemblies such as membrane proteins, large molecular machines, protein aggregates and surface bound molecules difficult using solution NMR methods unless ^13^C-methyl labeling, deuteration and TROSY methods are employed [191]. The decreased molecular tumbling rate of a protein when bound to a high MW complex leads to a large increase in the relaxation rate of the magnetization in the transverse plane, making these species invisible by classic NMR methods. However, this increase in the *T*_2_ rates of the bound molecule is the basis of the DEST experiment, recently proposed by Clore and co-workers [192]. The same basic concept has been used in the past to study protein–ligand interactions [193]. To understand these experiments, imagine a system where an NMR-visible state (e.g. a monomer) is in exchange with a species of high molecular weight (eg. an oligomer or an ensemble of oligomers, Fig. 8A), where the two states show the same chemical shift (for instance if the monomer conformation is unchanged in the oligomeric species). In such a case, the monomeric species is expected to give rise to narrow lines in its NMR spectrum (Fig. 8A and B). In contrast, the oligomers formed give rise to very broad lines and thus are NMR invisible. In a DEST experiment a weak saturating field *B*_1_ is applied far off-resonance from the sharp monomeric signal (Fig. 8C), leaving the resonances that are not in exchange with the species of interest unaffected. However, the extreme line-width of the bound resonances cause the saturation to be transferred through chemical exchange from the high molecular weight species to the NMR-visible state, resulting in an attenuation of the sharp signals that arise from the monomer (even when *B*_1_ is applied far off-resonance) (Fig. 8C). The observed attenuation depends on the *R*_2_ of the bound-invisible state and thus can give information on the dynamics of the ‘dark’ state. Much like the CPMG experiment, in DEST the invisible NMR state can be detected by its effect on the sharp lines of the visible species [143,194].

The DEST approach is ideally suited to studies of protein aggregation since the equilibrium between the low and high molecular weight species can be tuned by adjusting the experimental conditions (for instance protein concentration, temperature and pH) and the exchange rate is expected to be slow. One such study was performed on Aβ40/42 [194]. Although no chemical shift differences were observed between 50 and 270 μM Aβ, the *T*_2_ relaxation rates were found to be elevated by ~2 s^−1^ in the more concentrated sample [194,195]. Biophysical analysis (EM and AUC) showed that at high protein concentrations the peptide is in exchange with protofibrils, and DEST experiments were used to analyze this equilibrium. Global fitting of DEST and *T*_2_ relaxation data showed that a simple two state (monomer/fibril-bound) scheme is not sufficient to describe the experimental data. Instead, a pseudo-two state model had to be invoked [194]. In this model residues can be either in direct contact with the protofibril, or alternatively, are tethered to the protofibril indirectly by other regions of the same polypeptide chain that makes direct contact with the protofibril. Such an analysis yields the global *k*_on_ and *k*_off_ rates, as well as per residue tethering rates (*k*_3_), and the residue-specific *R*_2_ of the bound state, essentially providing a complete picture of the exchange event[194]. For both Aβ40/42 the N-terminal 8 residues were found to be tethered rather than directly bound, although differences between the two peptides were observed in the C-terminal region. Specifically, the C-terminal two residues of Aβ42 are mobile, while the C-terminus participates in the core of Aβ40 fibrils [194]. These differences may be the origin of the increased nucleation rates of Aβ42 [194].

In general, DEST-type experiments (now further developed for methyl bearing side chains [196]) promise to provide crucial information about oligomer formation and surface-catalyzed secondary nucleation in amyloid formation [10,11,197], phenomena that are vitally important in our quest to understand amyloid assembly in structural and energetic terms.

### 4.3. Visualization of transient protein–protein interactions on pathway to fibril assembly using paramagnetic NMR

**Fig. 9:**
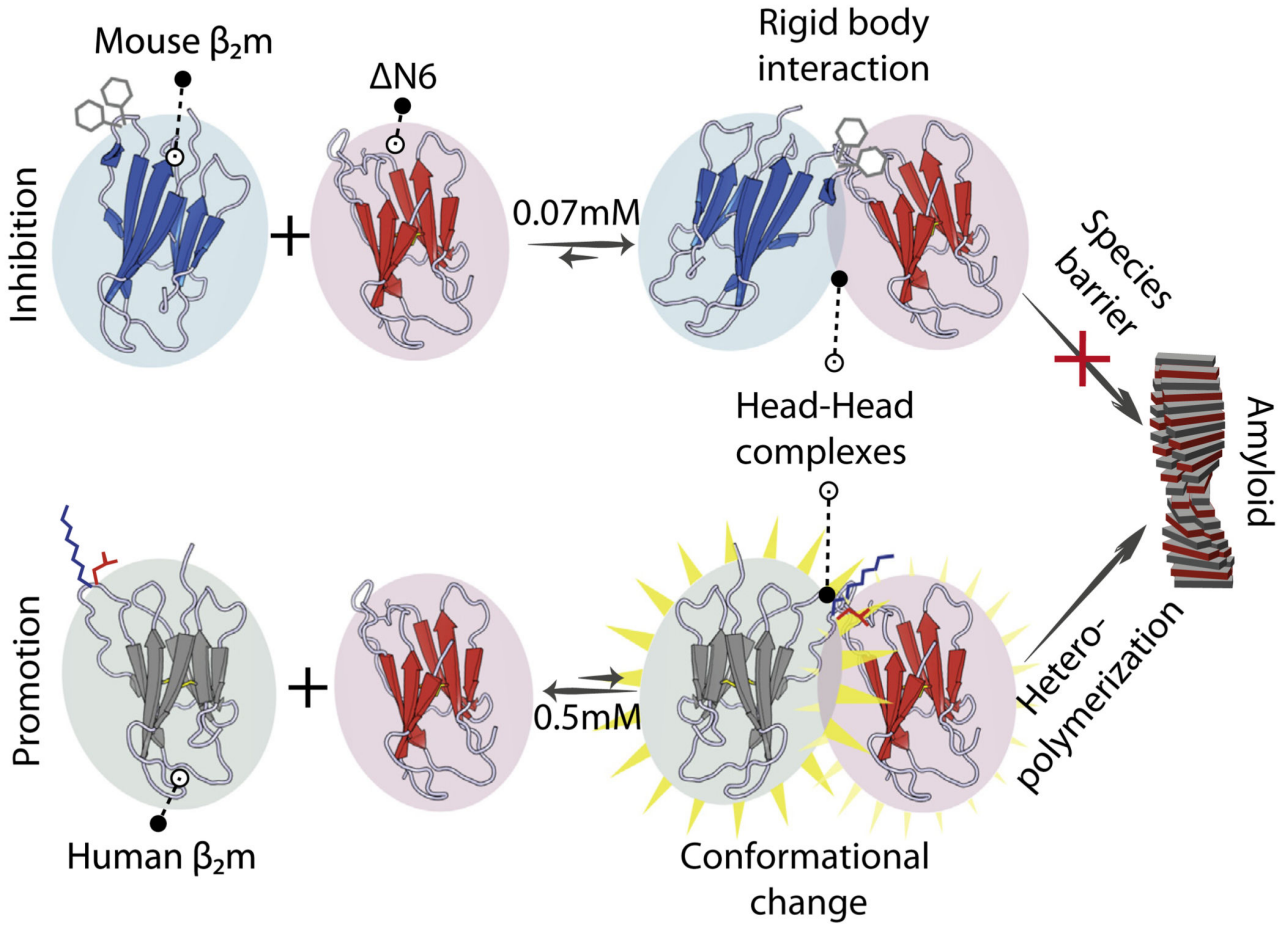
Transient protein–protein interactions in early amyloid assembly. Mβ_2_m binds ΔN6 to create a head-to-head ΔN6:mβ_2_m complex which is kinetically trapped and thus not aggregation-prone (top). Upon interaction the binding partners remain rigid (top). On the other hand the amyloid-promoting ΔN6-hβ_2_m association, although it involves similar binding surfaces, results in conformational changes in hβ_2_m, which is primed for amyloid formation (bottom). Reproduced from [84].

PRE methods provide sensitive probes able to detect heterogeneous and lowly populated species in solution (see Section 3.2). These studies can be used to analyze intra-molecular interactions by spin-labeling an NMR visible molecule (^13^C, ^15^N- or sparsely labeled) as in the case of IDPs (see Section 3.2), or by an inter-molecular interaction (by mixing a spin labeled but NMR-invisible (^12^C, ^14^N) molecule with an NMR-visible (^13^C, ^15^N) protein). In the latter case, the observed PRE effect measures protein–protein interactions, rather than intramolecular arrangements or motions. Intermolecular PRE studies, in combination with complementary NMR methodologies, have been used to study rare biomolecular interactions that lead to enhancement of the aggregation propensity of a poorly amyloidogenic protein (human β_2_m) by transient interaction with its aggregation-prone counterpart (ΔN6) [84]. Similar experiments were also used to probe the inhibitory ΔN6-murine β_2_m (mβ_2_m) association [84]. In these experiments, NMR-invisible spin-labeled amyloidogenic ΔN6 was mixed with NMR-visible less amyloidogenic variants of β_2_m (mβ_2_m or hβ_2_m), allowing identification of the interfaces involved in inhibition or promotion of assembly, respectively [84]. Unexpectedly, it was shown that the interaction surfaces are similar for each complex, showing that the progress of assembly is governed by the precise chemical details of the interface. Specifically, inhibition occurs via rigid body docking of monomers in a head-to-head orientation to form kinetically trapped dimers. By contrast, the promotion of fibrillation involves relatively weak protein association in a similar orientation but, in this case, biomolecular collision results in conformational changes in the initially non-fibrillogenic partner. Even subtle differences in the mode of interaction (even though its epicenter remains the same) can thus cause a vastly different outcome of the interaction that defines the course of amyloid assembly (Fig. 9).

Similar phenomena have been observed in prions or prion-like proteins, wherein very similar proteins from different species are either able to be converted into a prion-like state [9,198–201], or exhibit a species barrier [202–206]. Such systems, however, have yet to be studied in the detail achieved for the β_2_m assembly, by exploiting the power of paramagnetic NMR [84].

Inter-chain interactions between different monomers of α-synuclein have also been monitored using intermolecular PRE as described above [148]. Together, these reports demonstrate the power of modern NMR methods to shed light on important phenomena such as prion infectivity/transmissibility and prion species barriers. They can also be used to explain why some amyloid precursors co-assemble while others do not. This remains a significant unanswered question in the amyloid field, especially in the context of the recent appreciation that different amyloid sequences may co-assemble in amyloid disease [207].

### 4.4. H/D exchange in the fibrillar state

The wealth of hydrogen bonds that stabilize the cross-β fold of amyloid renders these assemblies ideal for H/D exchange studies. However, the high molecular weight of the fibrillar aggregates precludes direct observation of the species that are subject to H/D exchange by solution NMR (only extremely mobile regions of fibrils, usually in loops and/or in the N- or C-terminal regions, can be observed [208,209]). To deal with this issue various strategies have been proposed, with the most common being solubilization of the fibrils in an aprotic, organic solvent after H/D exchange has taken place. In these experiments, protonated fibrils are introduced into deuterated buffers and H/D exchange is allowed to proceed for a period of time. At specific time-points aliquots are centrifuged to pellet the fibrils. The pellet is then freeze-dried and re-suspended in a 95% d_6_-dimethyl sulfoxide (DMSO)-5% D_2_O solution to depolymerize the fibrils to monomeric subunits (DMSO is preferred as it does not contain any exchangeable protons). Analysis of each sample by 2D (^1^H-^15^N) NMR is then performed to determine *k*_obs(_*_j_*_)_ by fitting Eq. (10), and subsequently PF (Eq. (5)). Residues buried in the hydrophobic core of the fibril are expected to show a high PF in comparison with those that are not. Thus, DMSO quenched H/D exchange studies coupled with NMR spectroscopy represent a useful method to characterize fibril structure. Such studies have been performed on Aβ40/42, α-synuclein, β_2_m, transthyretin (TTR), HET-s and Sup35 and were used to identify the core and flexible regions of their fibrils. Importantly, H/D exchange studies are able to distinguish between different fibril types originating from the same protein precursor. Examples include β_2_m, and the SH3 domain (Table 1).

Apart from characterizing fibril structure, H/D exchange can be used to investigate the properties of intermediates formed during fibril assembly. Konuma et al. have used H/D exchange in combination with a stop-flow apparatus to study intermediates during fibril elongation of β_2_m at low pH (2.5) [233]. By carefully adjusting the pH of the reaction, H/D exchange or fibril elongation can be favored (competition H/D exchange) such that a per-residue image of kinetic intermediates formed during fibril elongation can be obtained. Such studies showed that fibril elongation commences by lateral association of an extended monomer conformation [233].

Amyloid fibrils are not static structures. Although the amyloidogenic proteins are trapped into a large macromolecular structure that appears stable thermodynamically and rigid macroscopically, each subunit can exhibit protein motions, not only in their side-chains, but also in their backbone atoms. In addition, fibrils are in equilibrium with monomeric or oligomeric species, and thus the model of H/D exchange for globular proteins presented in Section 2.3 needs to be reconsidered for such systems. Carulla et al. proposed a model for H/D exchange in which each fibril is in equilibrium between a non-exchangeable (*C_i_*)^(*j*,H)^ and an exchangeable form (*C_i_*)^j*,H)^ [234]. Once in the exchangeable form, all hydrogens exchange to deuterons with a first order rate constant *k*_ex(*j*)_, (Eq. (9)). The observed rate of exchange *k*_obs(*j*)_ is related to the observed rate of exchange of the monomer, *k*_obs(M)_, corrected by a protection factor PF_(_*_j_*_)_, to account for its incorporation into the specific fibril structure (Eq. (10)). The concentration of fully deuterated molecules at a given time into the H/D exchange experiments, increases exponentially as shown by Eq. (11).

(9) $${\left(C_{i}\right)}^{\left(j,H\right)}\overset{k_{\left(j\right)}}{\underset{k_{\left(j^{*}\right)}}{⇌}}{\left(C_{i}\right)}^{\left(j^{*},H\right)}\overset{k_{\mathrm{ex}(j)}}{\to }{\left(C_{i}\right)}^{\left(j,D\right)}$$

where (*C_i_*)^(*j*,*H*)^ and (*C_i_*)^(j*,H)^ represent the protonated exchangeable and non-exchangeable forms of the fibril, (*C_i_*)^(*j*,D)^ the deuterated fibrillar state. *k_(j)_* and k_(j*)_ are the forward and reverse rates for the (*C_i_*)^(*j*,*H*)^ to (*C_i_*)^(j*,H)^ transition and *k*_ex(*j*)_ is the rate of H/D exchange.

(10) $$k_{\mathrm{obs}(j)}=k_{\mathrm{obs}(M)}∕{\mathrm{PF}}_{\left(j\right)}$$

where *k*_obs(*j*)_, *k*_obs(M)_ are the observed H/D exchange rates for the fibril and monomer respectively and PF_(*j*)_ is the protection factor in the fibrillar state.

(11) $${\left(C_{i}\right)}^{\left(j,D\right)}(t)={\left(C_{i}\right)}_{\mathrm{tot}}^{\left(j\right)}[1-\exp(-k_{\mathrm{obs}(j)}t)]$$

This model can be used to explain processes such as fragmentation or molecular recycling through fibril ends. However, different two-state models have also been proposed to describe fibril elongation or species interacting with the fibrils [89,233].

## 5. Perspectives

Investigating the structural, kinetic and thermodynamic properties of the species formed during amyloid assembly in atomic detail is of crucial importance in our quest to understand the structural mechanisms of amyloid formation. NMR is the only technique capable of characterizing dynamic non-native states, as well as the transient and lowly populated species formed in the early stages of aggregation in atomistic detail. However, solution NMR suffers from its well-known molecular size limitation, which restricts its use (predominantly) to lower order species formed in the early stages of assembly. The use of specific amino acid precursors that result in sparse labeling of methyl groups of specific amino-acid precursors has be used recently to overcome these limitations[235]. As methyl groups have a shorter correlation time than the protein in which they are located, specific labeling in combination with transverse relaxation optimized techniques (Met-TROSY), can be used to investigate large macromolecular assemblies in solution, extending the range of structures detectable by solution NMR to ~1 MDa [192,236]. Such approaches have been successful in the characterization of large molecular machines [237–239], but have yet to be used to explore the structural properties of amyloid oligomers. The structural elucidation of oligomeric species can be greatly aided by PRE methods (discussed in Sections 3.2 and 4.2). Even though PRE techniques are well suited for the identification of interfaces, the extraction of exact distances in some cases is problematic due to the increased flexibility of the paramagnetic moiety [125]. The development of new paramagnetic tags may lead to better defined interatomic distances [134]. Recent advances in the field of computational biology also hold great promise for enhancing our understanding of amyloid formation. Molecular dynamics (MD) simulations can now be extended to the ms timescale [240], making the simulation of protein dynamics that drive the formation of amyloidogenic intermediates possible. The development of accurate docking algorithms is also helping to model the polymerization of these species restrained by the plethora of data now available from using different NMR techniques [241–243]. Restraints from EM, SAXS and mass-spectrometry (MS) can provide crucial additional structural information on the aggregation process [26,142]. Direct electron detection extends the resolution of EM to near atomic structures, while cross-linking the meta-stable species formed during assembly, followed by MS analysis, will aid the identification of interfaces in large macromolecular assemblies [244]. Double electron–electron resonance (DEER) electron paramagnetic resonance (EPR) can also be used to probe distances up to 5 or 10 nm between two paramagnetic centers. DEER restraints, therefore, are ideal to complement NMR restraints in MD simulations or structure calculation protocols [245]. A combination of solution-, solid-state NMR, EM, MS, EPR and also single molecule Förster resonance energy transfer (FRET) restraints together promise to shed light on the mechanisms of aggregation and to reveal why, and how, normally innocuous and functional proteins can convert into amyloidogenic conformers that self-assemble and cause devastating diseases.

## Figures and Tables

**Table 1:** H/D exchange studies on amyloid aggregates probed by solution NMR.

Amyloid precursor	Aggregation state	Reference
Aβ(25–35)	Fibril	[210]
Aβ(1–40)	Oligomers	[211]
Aβ(1–40) E22G	Fibrils	[212]
Aβ(1–42)	Fibrils	[213,214]
Acylphosphatase	Oligomers	[215]
α-Synuclein	Fibrils	[209]
α-Synuclein A30P	Fibrils	[216]
α-Synuclein (30–110)	Fibrils	[209]
β_2_m	Fibrils	[208,217–219]
Prion protein (106–126)	Fibrils	[220]
Prion protein (127–147)	Fibrils	[221]
RNAse A	Oligomers	[222]
SH3 domain	Oligomers	[223]
Cystatin	Fibrils/oligomers	[224]
Sup35	Fibrils	[225]
CspA	Fibrils	[226]
HET-s (218–289)	Fibrils	[227–229]
TTR	Oligomers	[230]
TTR (Y114C)	Fibrils	[231]
Bacterial inclusion bodies (BMP2 (13–74)^a^, ESAT-6^b^, MOG(ECD)^c^)	Fibrils	[232]

a β-sheet fragment residues 13–74 of the secretory human bone morphogenetic protein-2.

b Early secreted antigen 6-kDa protein.

c The extracellular domain (ECD) of the human membrane protein myelin oligodendrocyte glycoprotein.

## Glossary of abbreviations

Å: Ångström
AFM: atomic force microscopy
AUC: analytical ultracentrifugation
Aβ: amyloid-beta peptide
CD: circular dichroism
CPMD rd: Carr–Purcell–Meiboom–Gill relaxation dispersion
CSA: chemical shift anisotropy
DNA: deoxyribonucleic acid
DRA: dialysis-related amyloidosis
DEER: double electron–electron resonance
DEST: dark exchange saturation transfer
EM: electron microscopy
ESI-MS: electron-spray-ionisation mass spectrometry
EPR: electron paramagnetic resonance
FRET: Förster resonance energy transfer
FTIR: Fourier-transform infrared
H/D exchange: hydrogen–deuterium exchange
HSQC: heteronuclear singe quantum coherence
HMQC: heteronuclear multiple quantum coherence
*H*β_2_*m*: human β_2_m
IDP: intrinsically disordered protein
MAS: magic angle spinning
MDa: mega-Dalton
MHC-I: major histocompatibility complex-I
MTSL: S-(2,2,5,5-tetramethyl-2,5-dihydro-1H-pyrrol-3-yl-oxyl)methyl methanesulfonothioate
*M*β_2_*m*: murine β_2_m
MD: molecular dynamics
NMR: nuclear magnetic resonance
nOe: nuclear Overhauser effect
PBD: protein data bank
pH: decimal cologarithm of hydrogen
ppm: parts per million
PRE: paramagnetic relaxation enhancement
*R*_1_: longitudinal relaxation rate
*R*_2_: transverse relaxation rate
RDC: residual dipolar coupling
RMSD: root mean square deviation
SAXS: small angle X-ray scattering
SedNMR: sedimentation NMR
ssNMR: solid state NMR
*T*_1_: longitudinal relaxation time
*T*_2_: transverse relaxation time
TEM: transmission electron microscopy
TROSY: transverse relaxation optimized spectroscopy
TTR: transthyretin
*β*_2_*m*: beta-2 microglobulin
Δδ: chemical shift difference
